# Supplementary material for: Decoding fertility behaviour of married women in Zambia: A multipronged analysis of bio-demographic, socio-economic and intermediate factors
Source: PLOS Glob Public Health. 2025 Jan 8;5(1):e0004088. doi: 10.1371/journal.pgph.0004088 (PMC11709287; doi:10.1371/journal.pgph.0004088)
Supplement: Attachment — Submitted filename: Response to Reveiwers_Rebutal Letter_Editor PloS One.pdf [file pgph.0004088.s002.pdf]

Mulungushi University
P.O. Box 80415
Kabwe – Zambia

12th November, 2024

The Editor
PLOS One Global Health

Dear Editor,

Re: Rebuttal letter to the reviewer's comments

We are grateful to the reviewers for their thoughtful and insightful comments on our manuscript, *"Decoding fertility behaviour of married women in Zambia: A multipronged analysis of bio-demographic, socio-economic and intermediate factors."* We have carefully considered their feedback and made significant revisions to address their concerns.

Please refer to the summary below and the detailed attached reviewer response sheet for a detailed explanation of the changes made:

Reviewer 1

- The reviewer provided valuable feedback that significantly enhanced the manuscript. All of their comments were carefully considered and addressed through appropriate revisions.
- The reviewer requested that we provide details on the intermediate variables this has been done and highlighted in red in the last paragraph of page one and first and secondar paragraph of page two with appropriate in-text citations.
- Equally, the reviewer raised concerns about our discussion being too long (3 pages). This has since been reduced at least two pages and most of the points have been amalgamated to make it concise and succinct.

Reviewer 2

- All of the reviewer's concerns have been satisfactorily addressed, as outlined in the response sheet. In particular, a comprehensive explanation of why the Davis and Blake theory is more appropriate for our study unlike the more quantifiable Bongaarts Theory that has been widely used. This has been highlighted in red to make it clearer as to why the Davis and Blake intermediate variable suits this study
- A clarification on how the religion variable was classified has been provided in the explanatory variables and highlighted in red
- Their concern on the monotonous use of Zambia in the headings has been revised with the sections suggested that we do away with this adjusted accordingly.
- The concern on page 18 concerning lack of theoretical support for how the discussion point was crafted has been adjusted to make it clear. Importantly, the further analysis

has been suggested under further research of the conclusion which might be beyond the scope of this study.

- Importantly, the reviewer further requested that we revise the typos and some obvious errors in our manuscript. This has been done throughout the main document with revisions all highlighted in red.
- There is only one exception to what the reviewer had suggested, on the classification of variables which we have decided to keep based on previous similar studies that classified variables such as fertility preference under bio-demographic, and the rest under socio-economic characteristics.

As authors, we also wish to bring to the editors' attention that we have revised one word in the title from Multifaceted to Multipronged to align with the analytical procedures undertake in the analysis.

We believe that these revisions have significantly strengthened our manuscript. We are confident that the revised version now meets the high standards of your journal.

Thank you again for your time and consideration. We look forward to your decision.

Yours sincerely,

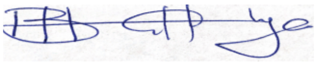

Bwalya Bupe Bwalya
Corresponding Author
