# Supplementary material for: Decoding fertility behaviour of married women in Zambia: A multipronged analysis of bio-demographic, socio-economic and intermediate factors
Source: PLOS Glob Public Health. 2025 Jan 8;5(1):e0004088. doi: 10.1371/journal.pgph.0004088 (PMC11709287; doi:10.1371/journal.pgph.0004088)
Supplement: Attachment — Submitted filename: Reviewer Comments (October 24, 2024)-by HatamHosseini.docx [file pgph.0004088.s001.docx]

**Comments to the Editor-in-Chief**

From 2012 to 2022, Zambia experienced a 17.7% decline in fertility, decreasing from 5.15 to 4.24 children per woman. Despite the global trend of falling fertility rates below replacement levels in numerous developing countries, Zambia's fertility rate remains high, with only a modest decline observed. Consequently, understanding the persistently high fertility levels in Zambia, in contrast to the significant declines seen in other African countries, is crucial. This article employs extensive large-scale data with a substantial sample size, enhancing the importance and generalizability of the findings. Analyzing fertility trends and the escalating annual population growth over the past decade underscores the critical need to address these issues in Zambia. Viewed from this perspective, the findings of this article merit serious consideration.

**Comments to the Author(s)**

1. The number of children ever born (CEB), as a longitudinal measure, reflects the lifetime fertility of women, unlike the total fertility rate (TFR), which is a cross-sectional measure. Consequently, it is expected that women who married at a younger age (before 18 in this study) or had a history of abortion, compared to those who married after 18 and did not experience an abortion, will exhibit higher fertility. This finding remains significant even after controlling for other explanatory variables.

2. Given the methodological issues associated with Davis and Blake's intermediate fertility variables and the introduction of the proximate determinants (PD) of fertility by John Bongaarts (1978) and Bongaarts and Potter (1983), it is recommended that the authors also refer briefly to the Bongaarts model. Moreover, it is necessary to explain that, considering the nature of the dependent variable (CEB) and its distinction from the total fertility rate (TFR), Davis and Blake's model has been employed as the theoretical framework for this research.

3. In the classification of control variables, I observe some interference in how the variables are grouped. For instance, sexual preference, religious affiliation, and the decision-making autonomy of women should be classified as cultural variables. Additionally, place of residence is a characteristic related to population distribution, and I propose that it be included among the demographic variables.

4. The variable of religious affiliation is divided into two categories: Catholic and others. It is necessary to specify which religious groups are included in the 'others' category.

5. This article explains fertility in Zambia, eliminating the need to repeat "Zambia" in some titles, such as the following:

- Bio-demographic, socio-economic, and intermediate correlates of children ever born in Zambia.
- Factors associated with children ever born in Zambia

6. Typing and writing errors are evident throughout the article, some highlighted in the PDF file. It is essential to edit the article carefully once more.

7. The analysis on page18 lacks theoretical support unless specific reasons are provided:

“On the contrary, women being poor can also make it difficult to afford raising children, with limited access to proper nutrition, healthcare, and education [68]. This can lead some women to choose to have fewer children. Therefore, the above demonstrates that the reasons behind demand for more children and poverty are multifaceted, and vary depending on cultural and social contexts.”

Typically, families living in poverty have a higher desire for children and an ideal number of children than those in better living conditions, as wealth flows from children to parents in poorer households. Further analysis is required.

Hatam Hosseini

October 24, 2024
